# Emotional arousal due to video stimuli reduces local and inter-regional synchronization of oscillatory cortical activities in alpha- and beta-bands

**DOI:** 10.1371/journal.pone.0255032

**Published:** 2021-07-23

**Authors:** Hyun Kim, Pukyeong Seo, Jeong Woo Choi, Kyung Hwan Kim

**Affiliations:** Department of Biomedical Engineering, College of Health Sciences, Yonsei University, Wonju, Korea; La Sapienza University of Rome, ITALY

## Abstract

The purpose of current study is to reveal spatiotemporal features of oscillatory EEG activities in response to emotional arousal induced by emotional video stimuli, and to find the characteristics of cortical activities showing significant difference according to arousal levels. The EEGs recorded during watching affective video clips were transformed to cortical current density time-series, and then, cluster-based permutation test was applied to determine the spatiotemporal origins of alpha- and beta-band activities showing significant difference between high and low arousal levels. We found stronger desynchronization of alpha-band activities due to higher arousal in visual areas, which may be due to stronger activation for sensory information processing for the highly arousing video stimuli. In precentral and superior parietal regions, the stronger desynchronization in alpha-and low beta-bands was observed for the high arousal stimuli. This is expected to reflect enhanced mirror neuron system activities, which is involved in understanding the intention of other’s action. Similar changes according to arousal level were found also in inter-regional phase synchronization in alpha- and beta-bands.

## Introduction

Emotional arousal refers to the state of heightened physiological activity, including fear and anger. The state of emotional arousal is highly related to cognitive factors such as attention and memory. Cuthbert et al. reported the correlation between attention and arousal levels while responding to affective pictures [1]. Memory enhancement was observed in response to the stimuli with high arousal [2,3]. Cortical activities due to arousal has been extensively studied using electroencephalogram (EEG) [4–6], and it has been consistently shown that the spectral powers of EEGs in alpha-band decrease in response to arousing stimuli [7–9]. However, contradictory results of alpha-band power increases due to arousal were also reported [10–12].

Most of these studies were performed with affective pictures, whereas video stimuli are more natural and effective to induce emotional arousal. The EEG studies on watching affective video have been rarely reported [13,14], and the characteristics of cortical responses to arousing video stimuli remain unclear. In this study, we tried to reveal spatiotemporal features of oscillatory EEG activities in response to emotional arousal induced by affective video stimuli. Specifically, we investigated the characteristics of cortical activities showing significant difference in EEG subband powers according to arousal levels. This was enabled by combining distributed cortical source model and cluster-based permutation test. In addition, we also explored the inter-regional synchronization/desynchronization of alpha- and beta-band activities. The use of video stimuli was effective since it provides an efficient way to induce emotional arousal, closer to real-world experiences than still image stimuli [15]. The results of this study may eventually contribute to develop a quantitative tool to evaluate the effectiveness of multimedia stimuli, by providing quantitative characterization of cortical responses.

## Methods

We recorded multichannel EEG signals while the subjects were watching emotional video stimuli. We developed an experimental paradigm that is similar to a real-world live video streaming environment to induce natural emotional responses. Time-frequency analysis and inter-regional functional connectivity analysis were performed, after cortical source reconstruction using distributed source model [16,17]. We used a data-driven approach to find out the features of cortical activities reflecting emotional arousal, based on nonparametric, cluster-based permutation test [18].

### Experimental methods

First, we selected 96 affective video clips (48 movie trailers, 48 TV series excerpts, 720p HD) as follows: One-minute length Korean movie trailer clips were selected from YouTube, watched over 1,000 times during 2013 to 2016. TV series excerpts with 2~3 min duration were selected from a Korean video streaming web service (Naver TV, http://tv.naver.com), if watched more than 10,000 times during 2013 to 2016. Then, they were cropped to 1 min duration around the main scene, by visual inspection.

**Fig 1:**
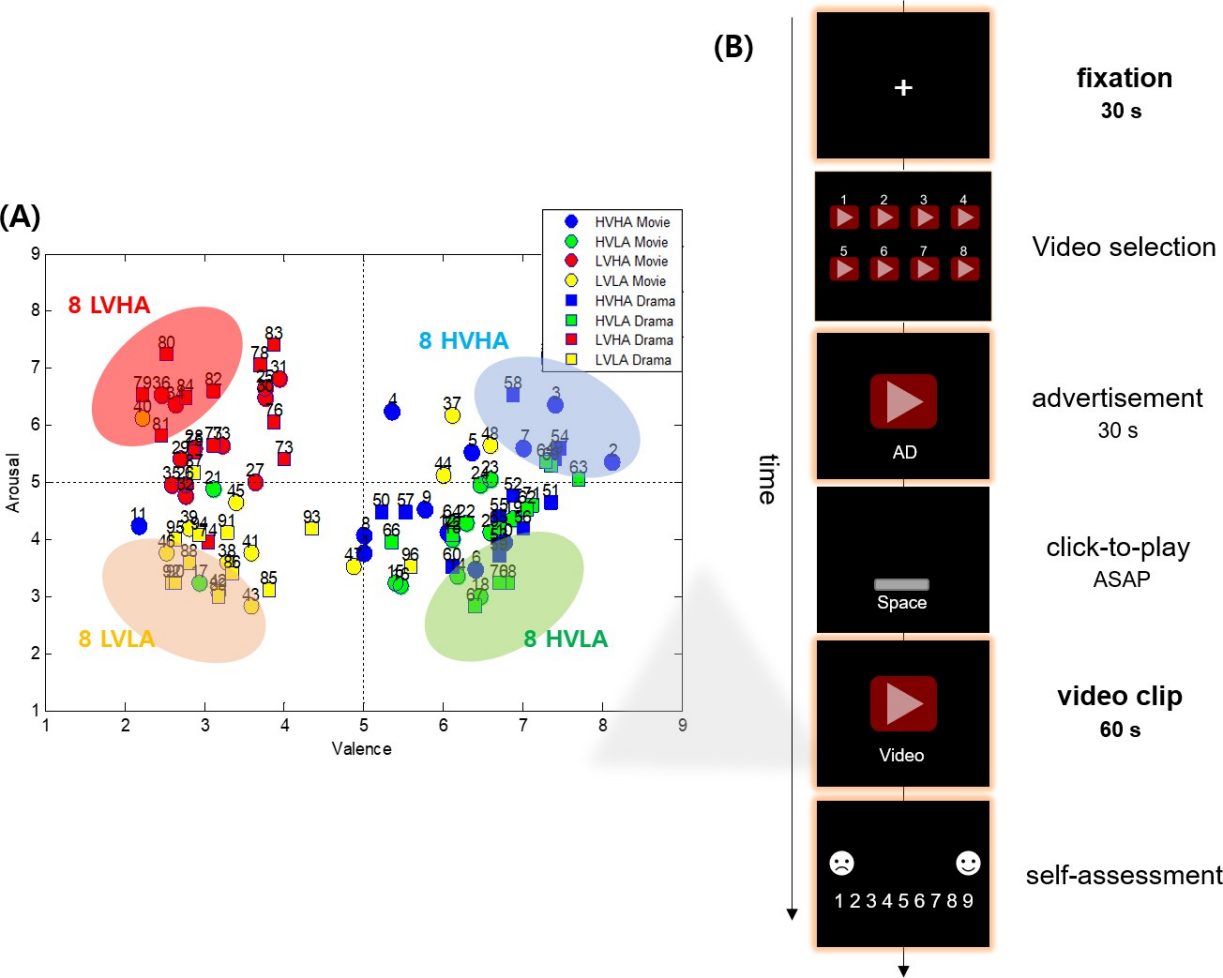
Experimental paradigm. (A) The distribution of the valence and arousal scores of video clips, averaged over all the participants of the pre-experiment. The finally selected video clips were indicated by shaded ellipses in the valence-arousal plane. (B) Temporal sequence of presenting visual stimuli in a single trial.

Before the main experiment, twenty-nine subjects (age: 23.2±1.8, 14 males, 15 females) were recruited for the pre-experiment to select the video clips. They assessed valence and arousal levels after watching each video, in 9-point Likert scale using self-assessment manikins (SAM) [19]. From the result of pre-experiment, thirty-two video clips (16 movie trailers, 16 TV series excerpts) were finally selected, by choosing the ones located far away from the x and y axes of the valence-arousal plane as shown in (Fig 1A). That is, the ones with extreme arousal and valence scores.

Twenty healthy university students were recruited for the main experiment (age: 23.6±1.8, 10 males, 10 females). The subjects were instructed to have enough sleep at the night before the experiment. Overall experiment was divided into four blocks, which consisted of eight trials of ~20 min duration as shown in (Fig 1B). Between each block, the subjects took a rest for ~5 min. Each trial comprised of fixation (30 s), video selection, advertisement (30 s), video presentation after confirmation (1 min), self-assessment (10 s), during which the subjects assessed the levels of valence, arousal, and liking in 9-point Likert scale. The video stimuli were presented by a stimulus presentation software (PRESENTATION; Neurobehavioral systems, Berkeley, CA, USA).

We recorded EEG signals at a sampling rate of 500 samples/s using BrainAmp EEG amplifier (Brain Products, GmbH, Munich, Germany), and an EEG cap with 64 Ag/AgCl electrodes placed according to the international 10–20 system (actiCAP, Brain Products, GmbH, Munich, Germany). The reference and ground electrodes were placed at FCz and AFz, respectively. The contact impedances were kept below 10 kΩ. The signals were recorded with a 60 Hz notch filter, 0.1 Hz highpass filter and 100 Hz lowpass filter. After downsampling (500→200 samples/s), stereotyped artifacts such as eye movement/blink or muscle activities were removed using independent component analysis (ICA, EEGLAB toolbox [20]). The study has been reviewed and approved by the institutional review board (IRB) of Yonsei University, Wonju campus (1041849-201501-BM-002-01 and 1041849-201804-BM-034-03). A written consent was obtained from all participants.

### Data analysis methods

**Fig 2:**
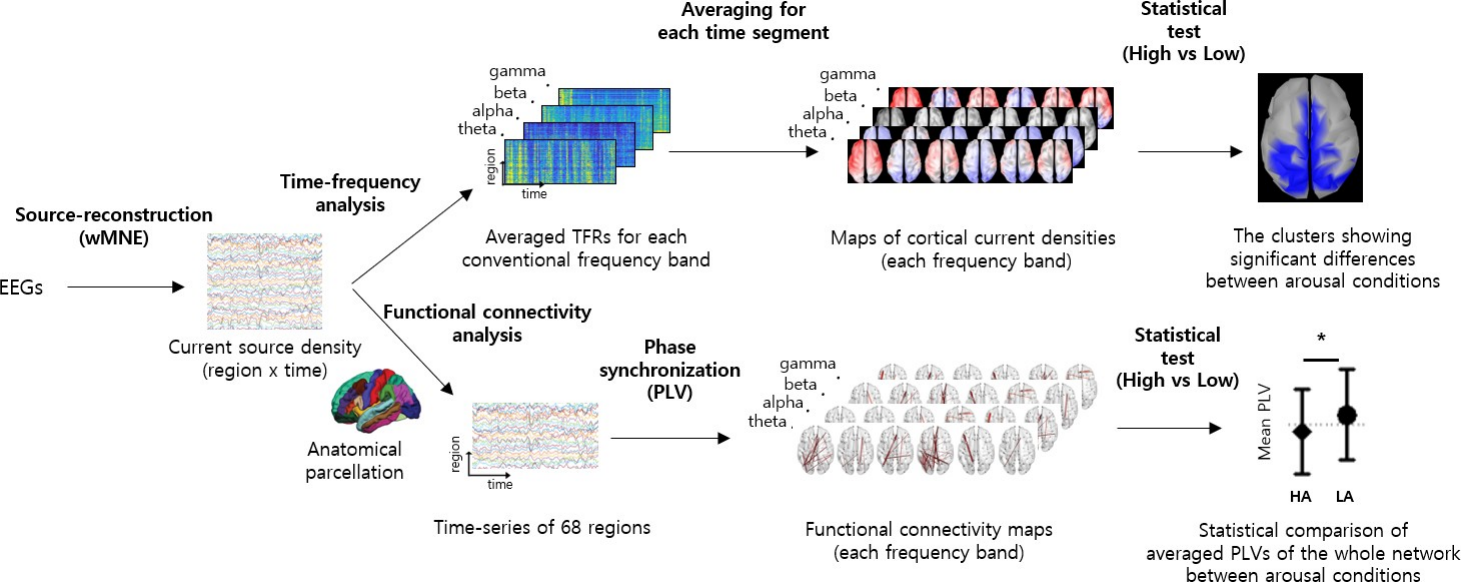
Procedure for EEG data analysis. The upper and lower parts of the Fig 2 present the procedures for the comparisons of cortical current source strength and inter-regional phase synchronization between high and low arousals.

Fig 2 illustrates overall procedure for EEG data analysis. The recorded EEG signals were divided into 90 s epochs (fixation: 30 s, video watching: 60 s). The signals were converted to cortical current source density time-series at 15,002 cortical points. Time-frequency analysis was performed to obtain the powers in alpha-, beta-, theta-, and gamma-bands at each 10 s temporal windows (theta: 4–7 Hz, alpha: 8–12 Hz, beta: 14–20 Hz, gamma: 30–50 Hz). For the functional connectivity analysis, debiased weighted phase lag index (dwPLI) was calculated in each frequency band within each 10 s temporal windows. The spectral powers and dwPLIs were statistically compared between high and low arousal conditions.

#### Cortical current source density estimation

An open source MATLAB toolbox, Brainstorm [17], was used to calculate the cortical current source density on the cortical surface. Boundary elements method (BEM) was used for the forward problem. The head model was computed with a symmetric BEM using OpenMEEG implemented in Brainstorm toolbox and the default ICBM152 template with 15,002 points on the cortical surface [21]. The current densities on cortical points were calculated by solving an inverse problem with weighted minimum norm estimation (wMNE) using Brainstorm toolbox [22].

#### Time-frequency analysis

To reduce the computational load of time-frequency analysis, 15,002 cortical points were downsampled to 500 points (by selecting the process “Less vertices” on the default anatomy in Brainstorm toolbox). And the current densities were projected onto the downsampled brain anatomy (by selecting the process “Project on default anatomy” in Brainstorm toolbox).

The temporal profiles of event-related spectral characteristics were examined using multitaper spectrum estimation (one second long Hanning window, 50% overlap) [23]. The time-frequency distribution of EEG power was calculated as the ratio of the relative power change compared to the baseline value at each frequency in the interval from 10–30 s, in order to reduce intersubject variability and to normalize power changes across different frequencies as follows:

$$E_{k}(f,t)=\frac{P_{k}(f,t)-R(f)}{R(f)}$$ (1)

Here *P_k_*(*f,t*) denotes the power of EEG for the *k*^th^ trial, estimated at time *t* and frequency *f*. *R*(*f*) denotes the averaged EEG power within baseline interval at each frequency *f*. The trial-averaged powers in four frequency bands were obtained for every 10 s interval.

#### Statistical comparisons

We adopted mass univariate analysis (MUA) for the comparison between two arousal conditions to search the cortical area showing significantly different activation [24]. First, 500 univariate comparisons by t-test were performed for 500 points on cortical surface. Then, the clusters of consecutive points showing high test statistic (t-value) were sought (thresholds: p = 0.0001 for alpha-band, and p = 0.001 for theta-, beta- and gamma-band). If more than five successive cortical points were selected, they were merged to form a cluster. We determined the threshold values for each subband differently so that the cluster size becomes less than 10% of all 500 cortical vertices. If the threshold was set to a specific value without considering the relative size of significant cluster, it occurred that too wide cortical region was determined to be significant, and thus, it became difficult to localize the crucial regions showing significantly different activation between conditions. For example, for alpha-band power, the threshold of t-value was set to smaller (0.0001 = 0.01%) since the clusters tended to be formed too wide.

A cluster was determined to be significantly different between arousal conditions, if its test statistic, the sum of t-values of all cortical points within the cluster, was higher than the threshold. The threshold was determined by surrogate data obtained from 10,000 random permutation of arousal conditions for each subjects [25], and was set to the highest 0.1% (corresponding to the significance level of 0.001) of the distribution of the test statistic for the surrogate data. FieldTrip toolbox was used for the statistical test [26].

#### Functional connectivity analysis

We conducted functional connectivity analysis on cortical current source time-series, instead of the scalp-level functional connectivity analysis between electrodes which causes poor reliability due to volume conduction/signal leakage [27]. The cortical surface was divided into 62 anatomical region of interests (ROIs) based on the Mindboogle atlas [28], and then, the current source time-series at the seed cortical point of each ROI was used to perform functional connectivity analysis. The instantaneous phases were calculated by Hilbert transform for the time-series of the four frequency bands (theta, alpha, beta, gamma).

Then, the dwPLI was calculated as a measure of functional connectivity between two cortical points for every 10 s interval for each trial. The dwPLI is a measure of phase synchronization which is weighted for nonzero lag phase activity and debiased for sample size. We adopted the dwPLI since it is known to be much less sensitive to the effect of volume conduction and leakage compared to the phase locking value [29].

To calculate dwPLI, the weighted phase lag index (wPLI) was calculated first as follows:

$$wPLI=\frac{\left|\frac{1}{n}\sum_{t=1}^{n}\left|I(S_{t})\right|*sgn(I(S_{t}))\right|}{\frac{1}{n}\sum_{t=1}^{n}\left|I(S_{t})\right|}$$ (2)

Here *n* denotes the number of time samples within each temporal window. $I(S_{t})$ indicates the imaginary part of the cross spectrum between two time-series at time point *t*. *sgn* () is signum function. Then, the dwPLI was obtained from the wPLI by debiasing using Vinck’s method [30]. To determine significance at each temporal window, the null distribution of dwPLI was estimated from 10,000 surrogate data, which were generated by randomizing phases of Fourier transform of the time-series [31]. A dwPLI was determined to be significant if it was above the highest 5% of the null distribution.

After subtracting and dividing the baseline values (similarly to the calculation of relative power change in Eq (1)), event-related dwPLI was obtained by averaging over trials. Consequently, positive and negative values of event-related dwPLI indicate the increase and decrease of phase locking during watching video clip relative to baseline, respectively. Paired sample t-test was used to compare the event-related dwPLI averaged over all possible pairs of cortical points (3,844 pairs, from the 62 regions) for each subject, to determine whether the functional connectivity was significantly different between high and low arousal conditions.

## Results

Significant differences of self-assessment scores were found between high and low arousal conditions (high: 5.88 ± 1.21, low: 3.33 ± 0.96, t-test, p<0.001), which implies that affective video clips successfully induced emotional arousal as we intended.

**Fig 3:**
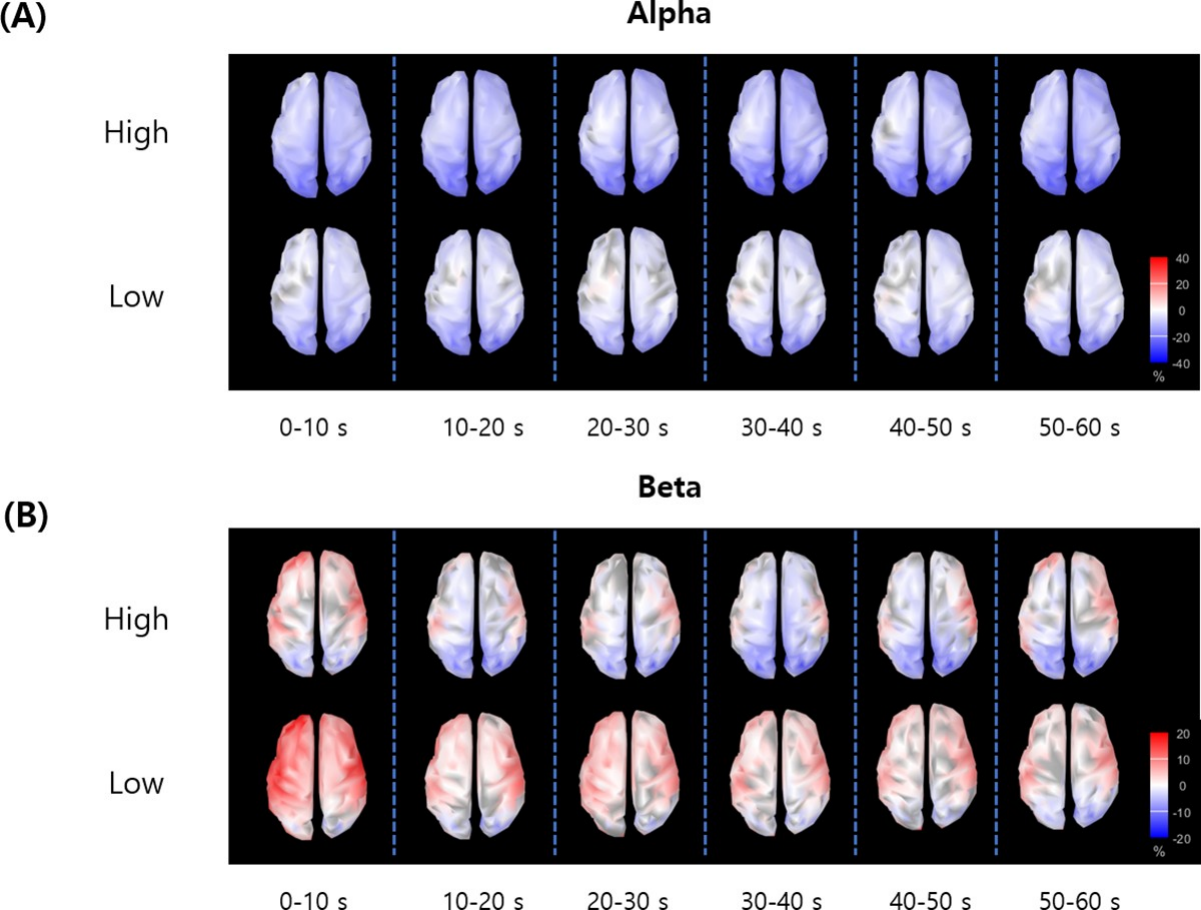
The distribution of spectral power changes over cortical surface at successive 10 s intervals. (A) Alpha-band and (B) beta-band.

Fig 3 shows the distribution of spectral power changes (alpha- and beta-bands) over cortical surface at successive 10 s intervals. The alpha-band power was decreased during watching video stimuli for both high and low arousal conditions, most strongly in visual areas (Fig 3A). For high arousal condition, the alpha-band power decrease was more widespread. The beta-band power was increased in prefrontal and temporal areas, while it was decreased in visual areas during video watching. Prominent decrease of beta-band power was observed over occipital and central regions especially in high arousal condition (Fig 3B).

**Fig 4:**
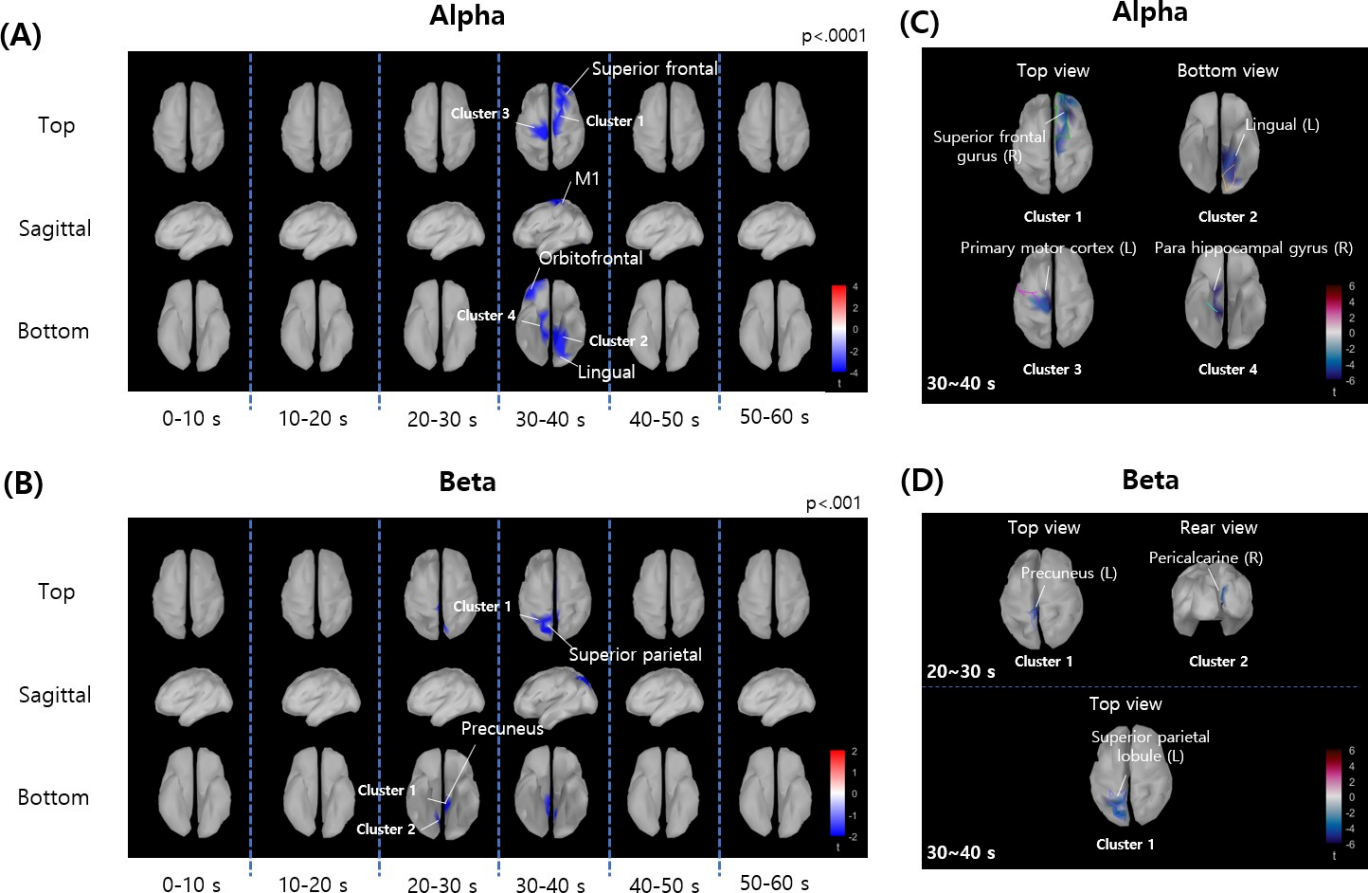
The distribution of t-values over cortical surface at successive 10 s intervals. The t-scores within the clusters showing significant difference between high and low arousal conditions were shown by color-codes for (A) alpha-band and (B) beta-band. In total, eight significant clusters were found. More detailed views of the seven significant clusters are depicted in (C) alpha-band and (D) beta-band, respectively.

The clusters of cortical points showing significant differences in spectral powers between arousal levels were found in alpha- and beta-bands. No significant clusters were found in theta- and gamma- bands. Fig 4 shows the distribution of t-values on cortical surface at each 10 s interval. The t-values above threshold were denoted by blue color (higher spectral power for lower arousal). Significant alpha-band power decreases for higher arousal were found most apparently during 30–40 s, in primary motor cortex, right lateral orbitofrontal, right superior frontal, and left lingual gyrus (Fig 4A). For beta-band power, significant decreases for higher arousal were observed mostly during 20–30 s and 30–40 s intervals, in superior parietal area and precuneus (Fig 4B).

We found four clusters of cortical points showing significant differences of alpha-band power between high and low arousal conditions (shown in Fig 4C). The largest cluster showing alpha-band power decrease for high arousal condition was found over right superior frontal gyrus in 30–40 s interval (cluster size: 19 cortical points, p<0.001). For the beta-band, we found three clusters showing significant decrease for the higher arousal condition (Fig 4D). The largest cluster was observed at left superior parietal lobule in 30–40 s interval (cluster size: 15 cortical points, p<0.001).

**Fig 5:**
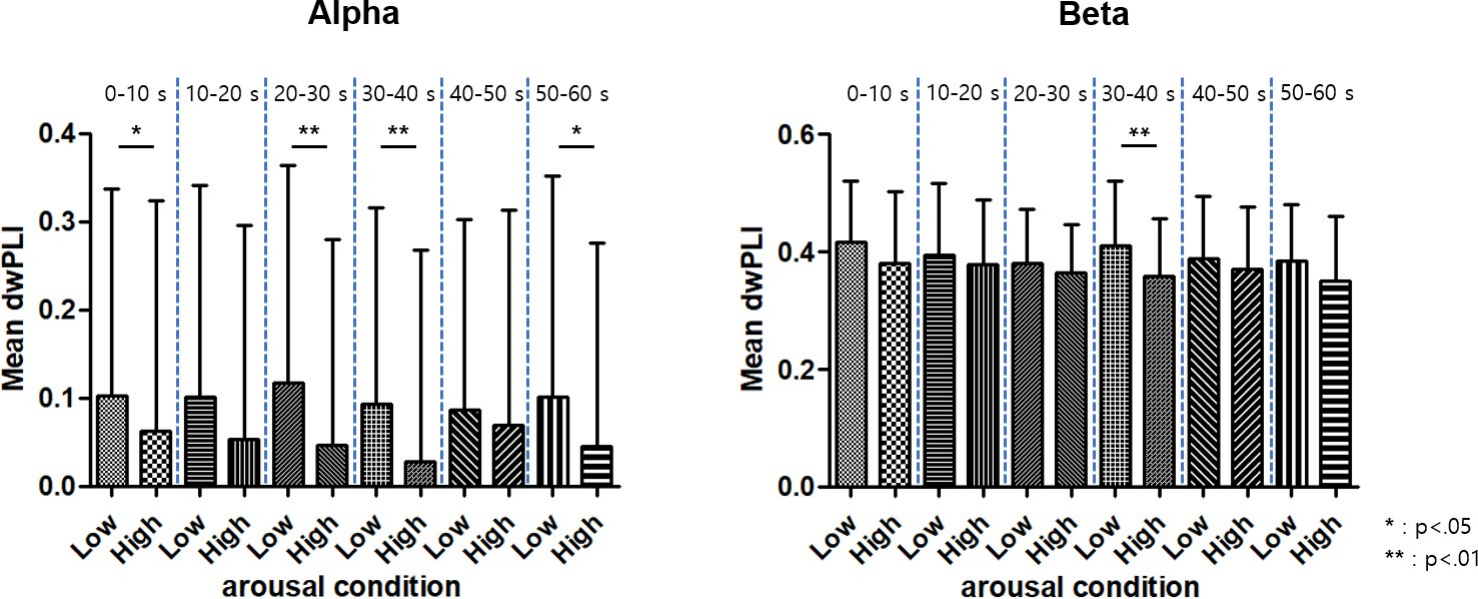
The average debiased weighted phase lag index of the whole network in each 10 s time interval for high and low arousal conditions (alpha- and beta-bands). Asterisks denote significant differences between arousal conditions (*: p<0.05; **: p<0.01).

Just as the spectral powers which are expected to represent local synchronization of neural activities, the inter-regional functional connectivity were decreased for high arousal. Fig 5 shows the averaged dwPLIs in alpha- and beta-bands in every 10 s interval. The dwPLIs in both alpha- and beta-bands were significantly decreased for higher arousal at 30–40 second intervals (p<0.05), when the significant differences of alpha- and beta-bands spectral powers were observed. The alpha-band dwPLIs were significantly decreased for higher arousal levels also at other 10 s intervals (p<0.01 for 20–30 s, p<0.05 for 0–10 s, 50–60 s). There was no significant difference between arousal conditions in theta- and gamma-band.

## Discussion

The purpose of this study was to reveal the neural correlates of emotional arousal induced by watching affective video clips. Our results showed that the EEG spectral power and phase synchronization in alpha- and beta-bands were significantly decreased during watching video clips with high arousal, compared to those with low arousal. Our findings may imply that highly arousing video triggers attentional information processing due to incoming visual stimuli, which is reflected in the desynchronization of oscillatory alpha- and beta-band oscillatory activities due to two different mechanisms.

We expect that the stronger desynchronization of alpha-band activities due to higher arousal in visual areas seems to reflect stronger cortical activation for sensory information processing, and is consistent with previous results [8]. The desynchronization of beta-band activities is expected to reflect mirror neuron system activities, which is also stronger for higher arousal.

An important contribution of this study is to characterize the effect of arousal on brain activities in response to emotional video stimuli. Our video stimuli include dynamic visual stimuli and auditory stimuli as well. We observed characteristic brain activities reflecting visual sensory and emotional information processing, which is differential from the responses to still image, although emotional video and still image induces some common cortical activities.

We showed that the alpha- and beta-bands powers and inter-regional phase synchronies are reduced during mid and late temporal periods of watching emotionally arousing video clips over ~1 min period, whereas the still image studies investigate neural activities within several seconds. Despite limitation in spatial resolution and precision, we also tried to reveal the locations on cortical surface showing characteristic changes by distributed source localization. We estimated that the characteristic changes in spectral power and functional connectivity due to arousing videos originate from attentional information processing and mirror neuron system activity.

### Alpha-band desynchronization for attentional processing

Alpha-band oscillation has been known to represent attentional inhibitory process which suppresses task-irrelevant information [12], and has been shown to be decreased in task-relevant regions, whereas increased in task-irrelevant regions [32,33]. This is also in line with our results of the alpha-band desynchronization (Fig 3A), which was focused in visual area. We judge that the stimuli with higher arousal yielded stronger alpha-band desynchronization (Fig 4A), and resulted in enhanced attentional information processing. Previous studies have shown that alpha-band event-related desynchronization in occipital and frontal area is induced in response to emotional picture stimuli [8]. This is in accordance with our results which demonstrated that alpha-band power is reduced in visual area around lingual gyrus and frontal area.

In addition, a number of studies have shown that inter-regional synchronization of alpha-band activities is also decreased due to enhanced attention in cognitive processing [34–36]. Hanslmayr et al. reported that the power and phase synchrony in alpha-band were decreased for enhanced sensory processing due to externally oriented states [36]. Consistently with our results on inter-regional functional connectivity, Cho et al. showed significant decrease of alpha-band phase locking value due to the higher arousal level of rituals [37].

### Alpha- and beta-band desynchronization and mirror neuron system

It is well known that alpha- and low beta-band rhythms are suppressed during action observation and execution [38,39]. Based on this, we estimate that the desynchronization of beta-band activities and that of alpha-band activities in motor-related area may reflect the activation of mirror neuron system (MNS) in response to the video stimuli.

We found that the alpha-band activities over primary motor cortex and beta-band activities in superior parietal region and precuneus were more reduced for higher arousal video in middle-late periods as shown in Fig 4. These results seem to be related to the activation of mirror neuron system, as previous studies suggested that suppression of spectral powers in these frequency bands is a sensitive marker of mirror neuron activity [40–42], which is involved in understanding the intention of other’s action [43]. In addition, it is known that the motion in visual stimuli influences emotional arousal [44,45], and thus, may have induced the activation of cortical areas underpinning action observation. Based on quantitative analysis of motions in video stimuli [46], we verified that the video clips with higher arousal level contained significantly greater amount of motion contents. Our results on the alpha- and low beta-band power reduction is also in line with a previous study which showed that understanding other’s behaviors or emotions yield power suppression in these frequency bands [47,48].

The alpha- and beta-bands event-related desynchronizations (ERDs) were attributed to the MNS activity, which is known to be related to the observation of movement. Considering that the MNS is also recognized to play a role in understanding other’s intention and higher cognitive function regarding emotional information processing [49–51], our observation of MNS activity change may reflect cortical activity for intention understanding triggered by movement observation, which resulted in emotional arousal. As far as we know, the involvement of MNS in neural response to emotional video and the interpretation of the meaning of this have not been presented in previous studies.

### Analysis methods

We determined to perform time-series analysis using a finite temporal window because neural response to emotional video stimuli should show dynamic characteristics with wax and wane during overall watching duration. The choice of 10 s window seems to be arbitrary, however, we judged that 10 s is long enough to observe change of cortical activity while short enough to track temporal variation. The point is on the use of time-varying analysis which enabled the tracking of dynamic neural activity change, not the exact size of temporal window. Compared to previous studies which reported the results obtained from an arbitrarily chosen temporal epoch [52,53], we expect that our method provides more valuable information. We have also verified that essentially same results were obtained when we varied the window size to 5 or 15 s.

### Further studies

One of the limitations of the current study is that 64-channel EEGs and standard brain template were used to estimate cortical source activity. The results on the location should be further verified with higher density EEG recording and more rigorous source estimation based on individual MRI, since EEG-based cortical source reconstruction suffers from limited spatial resolution and inaccurate localization. Another limitation is that the scope of this study is only to investigate neural representations for emotional arousal effect. The neural activities reflecting emotional valence or interaction effect between arousal and valence during watching emotional video remain unclear. The questions could be resolved by further studies.

## Conclusion

We tried to identify the characteristics of neural activities which show significant differences between high and low arousal during watching emotional videos, including temporal periods and the locations on cortical surface. The alpha-band power reduction due to higher arousal was observed mainly in right superior frontal, orbitofrontal and left lingual gyrus, and this may reflect higher cortical activation for attentional processing. The stronger alpha-band desynchronization in primary motor cortex and the stronger beta-band desynchronization in superior parietal area and precuneus for higher arousal may reflect the increase of mirror neuron system activities. As explained above, all these findings imply that the arousal levels of emotional video induce differentiated neural activities in the mirror neuron system and the cortical areas which are involved in attentional sensory information processing.
